# Camelina seeds and their glucosinolate, glucocamelinin, counteract severe fatty liver in rodents with monogenic obesity

**DOI:** 10.1186/s12263-026-00807-x

**Published:** 2026-06-06

**Authors:** Adam Jurgoński, Paulina M. Opyd, Dorota Napiórkowska, Bartosz Fotschki, Łucja Brzuzan

**Affiliations:** 1 InLife Institute of Animal Reproduction and Food Research, Polish Academy of Sciences, Trylińskiego 18 Str., Olsztyn, 10-683 Poland; 2 Department of Animal Nutrition, Feed Science and Cattle Breeding, University of Warmia and Mazury in Olsztyn, Oczapowskiego 5 Str., Olsztyn, 10‑719 Poland

**Keywords:** *Camelina sativa*, FGF21, Glucosinolates, NAFLD, MASLD, Oilseeds, THR-*β*

## Abstract

**Background:**

Obesity is associated with metabolic dysfunction-associated steatotic liver disease (MASLD), for which drugs and nutraceuticals are being developed. We investigated whether camelina seeds from the *Brassicaceae* family and their components can attenuate metabolic disorders in animal models of genetic obesity, including fatty liver.

**Methods:**

In two separate 5-week experiments, female obese (*fa*/*fa*) Zucker rats and female *db*/*db* mice were used. By appropriately incorporating camelina seeds or their oil into the diet, we investigated, in the rat model, the effects of the oil and non-oil fractions on intestinal and liver function, as well as on lipid metabolism. Subsequently, in a mouse model, oral administration of glucocamelinin, the main camelina glucosinolate, was performed to determine whether this group of compounds might be responsible for the observed beneficial effects.

**Results:**

Dietary camelina seeds counteracted liver hypertrophy and steatosis in obese rats, as confirmed by macroscopic and histological images, along with a several-fold lower content of total fat, triglycerides, and cholesterol (*p* < 0.01 for each). The rat experiment indicated that the non-oil fraction of camelina seeds was responsible for these beneficial effects. Oral glucocamelinin attenuated liver hypertrophy and steatosis in obese mice, as confirmed by macroscopic and histological images, along with considerably lower total fat (*p* < 0.05) and cholesterol (*p* < 0.01) contents. In both obese rats and mice, these and other beneficial effects were associated with alterations in the hepatic expression of genes crucial to lipid and glucose metabolism, including a higher expression of those encoding thyroid hormone receptor *β* (THR-*β*; *p* < 0.01 and *p* < 0.05) and fibroblast growth factor 21 (FGF21; *p* < 0.01 and *p* < 0.05).

**Conclusion:**

These findings indicate that camelina seeds may be considered both a dietary component and a source of bioactive glucosinolates relevant to MASLD. The mechanism behind the lipid-lowering effect of glucocamelinin may involve hepatic activation of FGF21 signaling through THR-*β*.

**Supplementary Information:**

The online version contains supplementary material available at 10.1186/s12263-026-00807-x.

## Introduction

Findings to date show that common polygenic obesity and rare, severe, early-onset monogenic obesity share genetic and biological underpinnings, with the brain playing a key role in controlling energy intake and body weight [1]. Regardless of genetic background, one of the most common complications in overweight and obese adults is metabolic dysfunction-associated steatotic liver disease (MASLD), with an estimated global prevalence rate of 51% in this group of people [2]. The only treatment method available for this type of fatty liver is lifestyle changes related to diet and weight loss, which, in the most severe cases, are combined with bariatric surgery [3]. Thus, intensive research is underway on potential nutraceuticals and drugs that could prevent and treat this disease [4, 5].

Our previous research and others have shown that regular consumption of less common oilseeds (hempseed, flaxseed, chia seed) improves intestinal and liver disorders and lipid metabolism in experimental obesity of various etiologies [6–9]. We observed that the beneficial effects of certain oilseeds depend, to varying degrees, on the presence of polyunsaturated fatty acids (PUFAs), dietary fiber, and biologically active compounds specific to a given type of seeds [7, 8]. These results became the basis for intensifying further research to find the most beneficial oilseeds that could improve digestive and metabolic functions in the body. Here, we verified whether camelina (*Camelina sativa* (L.) Crantz) seeds can meet these needs and which components are responsible for potential benefits of their consumption.

Camelina, also called false flax, is a flowering plant in the family *Brassicaceae* and is occasionally cultivated in Europe and North America for its relatively high oil content (approx. 35%) [10]. Camelina seeds are rich in nutrients, including PUFAs, protein, fiber and biologically active compounds, especially glucosinolates [10, 11]. Their oil contains a relatively large amount of *α*-linolenic acid (35% or above), an *n*-3 fatty acid essential for our body, the content of which is usually insufficient in the daily diet [12]. Glucosinolates, in turn, are secondary plant metabolites and derivatives of glucose and amino acids specific to the *Brassicaceae* family containing both sulfur and nitrogen [13]. The safety of their consumption remains unclear, ranging from a proven goitrogenic effect [14] to recent evidence supporting their protective role in chronic diseases, including cardiovascular disease and cancer [15]. The study by Meadus et al. [16] indicated that camelina meal, a by-product of oil pressing rich in glucosinolates, especially glucocamelinin, can affect liver function in weaned pigs by activating genes responsible for xenobiotic metabolism. A recent study by Zhu et al. [17], in turn, showed that a glucosinolate extract from radish seeds can attenuate high-fat diet-induced obesity in mice by reducing lipid deposition in the liver and epididymal adipocytes, suggesting an important role of the gut microbiota as a mechanistic factor.

We hypothesized that regular ingestion of camelina seeds can attenuate digestive and metabolic disorders associated with monogenic obesity, including fatty liver, and that seed components such as PUFAs, fiber, and glucosinolates may be responsible for these effects.

## Materials and methods

### Camelina seeds, camelina seed oil and the glucocamelinin preparation

Whole camelina (*Camelina sativa* (L.) Crantz) seeds were purchased from Skolej (Kościelec, Kujawsko-Pomorskie Province, Poland), while unrefined, cold-pressed camelina seed oil was obtained from Olvita L.P. (Marcinowice, Dolnośląskie Province, Poland). Both companies specialize in the production of cold-pressed vegetable oils. The chemical composition of camelina seeds was quantified in triplicate by an accredited testing laboratory (Nuscana, Mrowino, Poland) in accordance with the Polish-European ISO standards, official procedures of AOAC or internal procedures using commonly known methods. Briefly, the dry matter and ash content were determined by the gravimetric method after drying the whole camelina seeds at 105 °C and 525 °C (PN-EN 1135:1999). The total dietary fiber was determined by the enzymatic-gravimetric method (AOAC 991.43:1994). Crude protein was determined by the Kjeldahl method (PN-EN ISO 20483:2014-02), and crude oil was determined by the Soxhlet extraction method. The nitrogen-free extract was then calculated by subtracting water, ash, fiber, crude protein and crude fat from 100. The fatty acid profile of the oil fraction extracted from the seeds and the oil itself was determined by gas chromatography with flame-ionization detection after previous conversion of the fatty acids into their respective methyl esters (PN-EN ISO 12966-1:2015 and 12966-2: 2011). The chemical composition of camelina seed oil and camelina seeds is shown in Table 1.

**Table 1:** Chemical composition of camelina seed oil and camelina seeds^1^

	Camelina seed oil^3^	Camelina seeds
Dry matter, % of fresh matter	–	91.2 ± 0.10
Ash, % of fresh matter	–	4.18 ± 0.01
Dietary fiber, % of fresh matter	–	25.8 ± 0.20
Nitrogen-free extract, % of fresh matter	–	0.02 ± 0.00
Crude protein, % of fresh matter	–	25.6 ± 0.10
Crude oil, % of fresh matter	–	35.6 ± 0.26
Fatty acids (% of oil)		
Palmitic acid (16:0)	5.08 ± 0.02	5.59 ± 0.02
Stearic acid (18:0)	2.21 ± 0.01	2.20 ± 0.01
Oleic acid (18:1 *n*-9)	12.2 ± 0.06	12.0 ± 0.03
Vaccenic acid (18:1 *n*-7)	1.06 ± 0.01	1.10 ± 0.01
Linoleic acid (18:2 *n*-6)	17.4 ± 0.01	17.0 ± 0.01
Arachidic acid (20:0)	1.37 ± 0.00	1.32 ± 0.01
* γ*-Linolenic acid (C18:3 *n*-6)	0.12 ± 0.00	0.14 ± 0.00
Gondoic acid (20:1 *n*-9)	12.8 ± 0.03	13.2 ± 0.03
* α*-Linolenic acid (18:3 n-3)	35.1 ± 0.05	33.9 ± 0.01
11,14-eicosadienoic acid (20:2 *n*-6)	1.82 ± 0.01	1.91 ± 0.01
Behenic acid (22:0)	0.31 ± 0.00	0.30 ± 0.00
Erucic acid (22:1 *n*-9)	2.83 ± 0.01	3.14 ± 0.01
11,14,17-eicosatrienoic acid (20:3 *n*-3)	1.36 ± 0.01	1.42 ± 0.01
13,16-docosadienoic acid (20:2 *n*-6)	0.18 ± 0.00	0.22 ± 0.00
Lignoceric acid (24:0)	0.17 ± 0.00	0.19 ± 0.00
Nervonic acid (24:1 *n*-9)	0.62 ± 0.01	0.67 ± 0.00
Other (unidentified)	0.48 ± 0.00	0.54 ± 0.00
SFAs^2^	9.14 ± 0.02	9.60 ± 0.02
MUFAs^2^	29.5 ± 0.02	30.1 ± 0.02
PUFAs^2^	56.0 ± 0.03	54.6 ± 0.01
* n*-3	36.5 ± 0.03	35.4 ± 0.01
* n*-6	19.5 ± 0.01	19.2 ± 0.01

^1^Values are means ± SDs (*n* = 3)

^2^MUFAs, monounsaturated fatty acids; PUFAs, polyunsaturated fatty acids; SFAs, saturated fatty acids

^3^Composition already published^[18]^

Glucocamelinin (10-methylsulfinyldecyl-glucosinolate) potassium salt preparation was purchased from Cfm Oskar Tropitzsch Ltd. (Marktredwitz, Bavaria, Germany). The batch had a purity of 98.3%, which was confirmed by a high-performance liquid chromatography (HPLC) method (column type: C-18-AQ 120-5µ, 250 × 4.6 mm; column temp.: 20 °C; mobile phase: acetonitrile/0.1% trifluoroacetic acid – 20/80, v/v; flow rate: 1 mL/min; detection at 225 nm).

### Ethics and other statements

The research was based on two experiments: one conducted on obese (*fa*/*fa*) Zucker rats and the other on obese (*db*/*db*) mice, both housed in the animal facility of the InLife Institute. The animals were used as an experimental model of monogenic obesity and advanced fatty liver, and prior to the experiments, they underwent a one-week acclimatization period to the experimental environment. Both experiment protocols were in accordance with European Union legislations for the care and use of laboratory animals (Directive 2010/63/EU) and ARRIVE guidelines, and were approved by the Local Institutional Animal Care and Use Committee in Olsztyn, Poland (permission numbers: 37/2017 and 75/2022 for rats and mice, respectively). The protocols were prepared prior to the experiments but were not registered in any online database. There were no specific criteria established or applied for including or excluding animals during the experiments and analyses. The animals were assigned to experimental groups based on their initial body weight, and potential confounders were minimized by randomly placing the cages in the experimental room. All individuals involved in the experiment were aware of the group allocation at each stage, but only the first author of the paper and the person responsible for preparing the experimental diets and preparations were aware of the specific experimental factor assigned to each group.

### Experiment 1 in rats

**Fig. 1:**
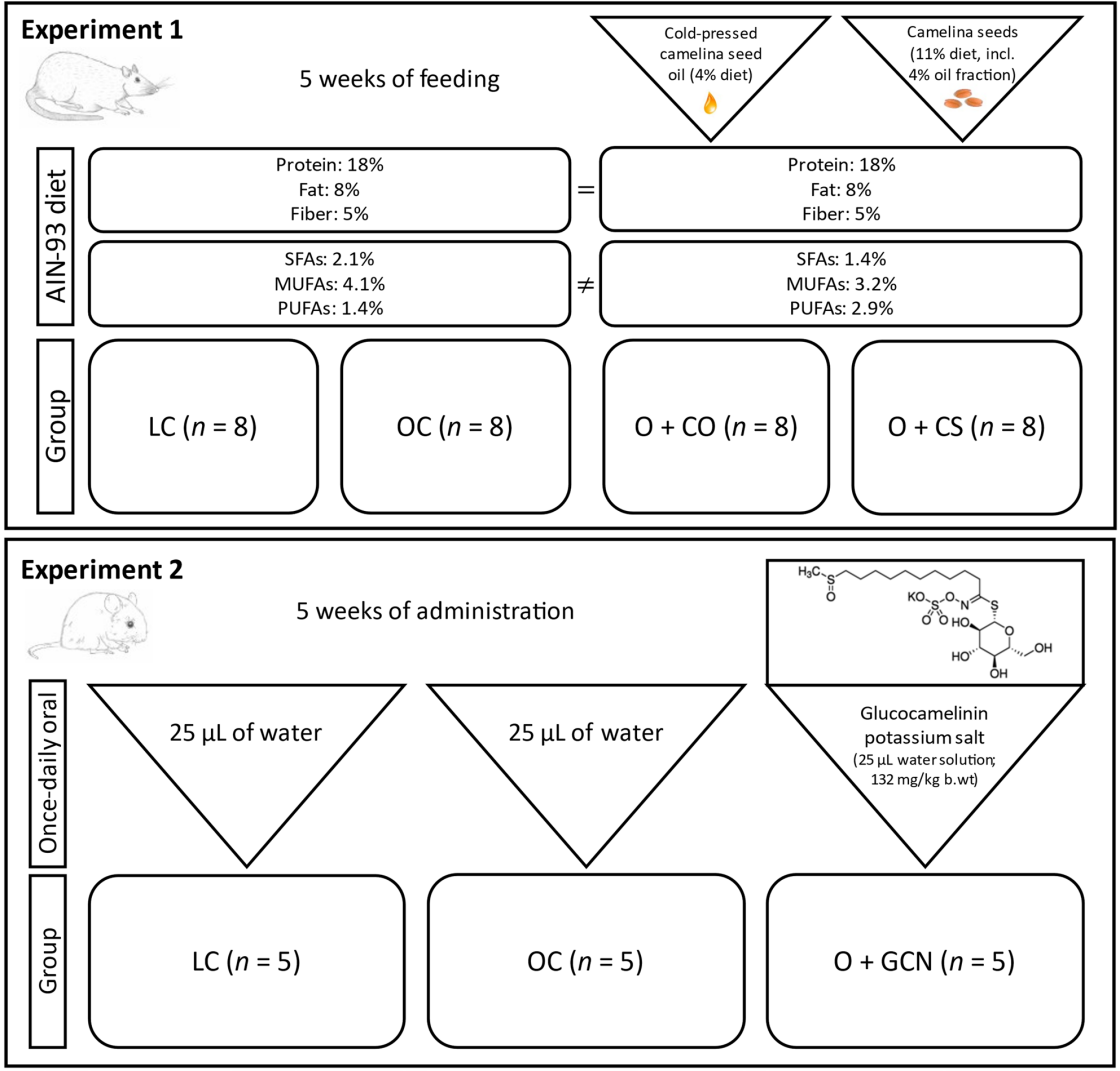
Design of Experiment 1 in lean and obese (*fa/fa*) Zucker rats and Experiment 2 in lean and obese (*db/db*) mice. LC, lean control; OC, obese control; O + CO, obese fed a diet containing camelina seed oil; O + CS, obese fed a diet containing camelina seeds; O + GCN, obese ingesting glucocamelinin. MUFAs, monounsaturated fatty acids; PUFAs, polyunsaturated fatty acids; SFAs, saturated fatty acids

The feeding experiment was conducted on lean (*Fa/fa*) and obese (*fa/fa*) female Zucker rats (32 in total) allocated into one lean and three obese groups of eight animals each and fed for 5 weeks with semipurified diets. The rats were approximately 6 weeks old and their body weights are shown in Supplementary Table 2. Each group was then fed a modified version of the semipurified casein diet recommended for rodents by Reeves [19]. The lean control (LC) group and obese control (OC) groups were fed a diet containing, among other components, casein, cellulose, and rapeseed (canola type) oil and palm oil (4% each) as sources of protein, fiber and fat, respectively. The other two obese groups were fed a modification of this diet, in which camelina seed oil was added at the expense of palm oil (4% diet; O + CO group) or ground camelina seeds were added at the expense of casein, cellulose and palm oil (11.23% diet; O + CS group). All diets had the same proportion of protein (18%), fat (8.3%) and fiber (5%), but the diet of the O + CS group had slightly lower carbohydrate content due to differences in corn starch content and the chemical composition of the camelina seeds. The percentage of oil derived from the tested seeds was the same in the diet containing ground camelina seeds and in the diet with their cold-pressed oil (4%), with the oil fraction in both cases replacing palm oil (a saturated fat source) in order to enhance the visibility of its biological effects. Diets fed to the O + CO and O + CS groups had also similar fatty acid profiles, including a more than two-fold increase in the proportion of PUFAs that originated either from camelina seed oil or camelina seeds. Thus, we were able to directly assess the effects of the oil fraction of camelina seeds, and indirectly, by comparing the obtained results, the effects of their non-oil fraction on the development of metabolic disorders. A similar experimental design has been used previously with other seed types [7, 8]. The detailed composition of the diets, which were freely available to rats for the entire experimental period, is shown in Supplementary Tables 1, and the design of Experiment 1 is shown in Fig. 1. After preparation, the diets were stored at 4 °C under limited access to oxygen for a maximum of one week. The rats were individually housed in plastic cages with environmental enrichments and maintained under controlled conditions (12 h light/dark cycle, temperature 21 ± 1 °C, relative humidity 55 ± 10%, and 15 air changes per hour).

### Experiment 2 in mice

In this experiment, lean (*db*/*+*) and obese (*db*/*db*) female mice were allocated into one lean and two obese groups of five animals each (15 in total). The mice were approximately 6 weeks old, and their body weights are shown in Supplementary Table 5. All groups were fed a standard pelleted mouse diet throughout the experiment. For 5 weeks, 25 µL of water was administered by gavage once daily at 10:00 AM to the lean control (LC) and obese control (OC) groups, whereas the remaining obese group received 25 µL of an aqueous solution of the glucocamelinin preparation using the same procedure (O + GCN group). The daily dose of glucocamelinin (132 mg/kg initial body weight) was set to match the intake of glucosinolates from seeds in Experiment 1. Glucosinolate intake was estimated based on previously published detailed regional data on their content in seeds (25.1 µmol/g dry weight) [20]. The design of Experiment 2 is shown in Fig. 1. The mice were housed in group-specific cages with environmental enrichments and maintained under controlled conditions (12 h light/dark cycle, temperature 21 ± 1 °C, relative humidity 55 ± 10%, and 15 air changes per hour).

### Sampling and analysis of biological material

At the end of Experiments 1 and 2, the animals were fasted and anaesthetized with a mixture of xylazine and ketamine in physiological saline (10 and 100 mg/kg body weight, respectively). Each animal was then weighed, and the abdomen was opened. Blood was collected from the *vena cava* (rats) or the heart (mice) into heparinized tubes. The blood was then centrifuged for 10 min at 380 × g and 4 °C, and the obtained plasma was frozen until analysis. The small intestine, cecum, liver, heart, and kidneys were then removed, weighed and frozen in liquid nitrogen or used for further procedures.

The mucosal disaccharidase activities (sucrase, maltase, and lactase) were measured according to the Dahlqvist method [21] with previously published modifications [6]. Samples of fresh cecal digesta were collected, and their pH values were measured using a microelectrode and pH/ION meter (Model 301, Hanna Instruments). The ammonia concentration in the fresh cecal digesta was determined after extraction and trapping in a boric acid solution, followed by direct titration with sulfuric acid in Conway dishes according to the method described by Hofirek and Haas [22]. The short-chain fatty acid (SCFA) concentrations were determined in the cecal digesta after storage at ‒20 °C using a gas chromatograph (Shimadzu Co.) and a capillary column (SGE BP21, 30 m × 0.53 mm; SGE Europe Ltd.) as previously described [23].

Liver lipids were extracted according to the method of Folch et al. [24] with previously described modifications [6]. Briefly, a piece of liver was homogenized with a 2:1 chloroform–methanol mixture using a homogenizer (IKA T25, USA) followed by centrifugation at 10,000 × g for 10 min. The supernatant was washed with distilled water, vortexed, and centrifuged for 15 min (5,000 × g). After removal of the upper phase, the lower phase containing lipids was evaporated under a nitrogen stream at 37 °C and weighed. The lipid fraction obtained in this way was then used for the spectrophotometric determination of cholesterol and triglyceride concentrations, using reagents from Alpha Diagnostics Ltd. (Warsaw, Poland).

The plasma cholesterol (total and its HDL fraction), triglycerides, glucose and bilirubin concentration, as well as the plasma aspartate transaminase (AST), alanine transaminase (ALT) and alkaline phosphatase (ALP) activity were determined using a biochemical analyzer (Pentra C200, Horiba Ltd., Japan). The total plasma concentration of bile acids was determined using a kit from Cell Biolabs kit (San Diego, CA, USA). The plasma insulin concentration was determined using an enzyme-linked immunosorbent assay kit manufactured by Demeditec Diagnostics GmbH (Kiel, Germany).

### Body composition analysis

At the beginning and end of Experiment 2, the body lean and fat masses of the mice were determined by time-domain NMR using the Minispec LF 90II analyzer (Bruker, Karlsruhe, Germany). The body lean and fat percentages were then calculated based on the initial and final body weights of the animals. The method relies on transmitting various radio frequency pulses into soft tissues to reorient the nuclear magnetic spins of the hydrogen and then detecting radio frequency signals generated by the hydrogen spins from these tissues. The contrast in relaxation times of the hydrogen spins found among adipose tissue and other soft tissues is then used to estimate their masses within the body.

### mRNA quantification

Gene mRNA expression levels in the small intestine and liver were quantified according to a previously described method [6] on equipment and reagents from Thermo Fisher Scientific (MA, USA). Briefly, RNA was extracted from the intestine and liver using TRI Reagent solution according to the manufacturer’s instruction. RNA quantity and quality were measured spectrophotometrically using a NanoDrop1000 and agarose gel electrophoresis, respectively. cDNA was synthesized from 500 ng of RNA using a High-Capacity cDNA reverse transcription kit with a ribonuclease inhibitor. The *β*-actin gene (Actb) was selected from three reference genes. The mRNA level of each tested gene was determined using species-specific and recommended TaqMan gene expression assay probes. Amplification was performed using the 7900HT Fast Real-Time PCR System under the following conditions: initial denaturation for 10 min at 95 °C; 40 cycles of 15 s at 95 °C and 1 min at 60 °C. Each run included a standard curve based on aliquots of pooled intestinal or liver RNA. All samples were analyzed in duplicate, and the mRNA levels were normalized to the reference gene.

### Liver histology

Liver sections were flushed with PBS and dried, and then immersed for 7 days in a 10% buffered formalin solution. After the formalin treatment, the sections were embedded in paraffin, and the obtained blocks were cut into 5-µm-thick sections using a Reichert microtome. The sections were dewaxed by immersing in xylene and dehydrated in an ethanol gradient solution. The pieces were then stained with hematoxylin and eosin (Merck, Darmstadt, Germany), according to the method described by Fischer et al. [25], and visualized using a light microscope connected to a digital camera and a computer image analysis program (Olympus Co., Tokyo, Japan).

### Statistical analysis and results presentation

The sample size was determined based on a previous experiment with a similar design and endpoints [7], as well as practical considerations, including ethical guidelines (both experiments) and the availability of the experimental factor (Experiment 2 in mice). A priori power analysis was performed assuming two effect sizes: 1.20 and 1.79 (Cohen’s d), a significance level of *α =* 0.05, and statistical power of 0.8. Taking into account both effect sizes, the mean number of rats sufficient to detect significant differences in the primary outcome measure was established at 8 per group. For mice, only the higher effect size was considered due to limited glucocamelinin availability (5 per group). The primary outcome measure used to determine the effect sizes and sample sizes was hepatic fat content in genetically obese rats (one control group vs. two experimental groups) [7], as it is a direct and quantifiable marker of liver steatosis. This variable was selected based on its relevance to the research objectives and its previously established sensitivity to a similar dietary intervention.

In Experiment 1 and 2, the Student’s *t*-test was used to determine significant differences between the lean control (LC) and obese control (OC) groups, whereas in Experiment 2, this test was also applied to determine differences between the two obese groups, including the group ingesting glucocamelinin (O + GCN group). If the data were not normally distributed according to the Shapiro-Wilk test or the variance was not homogeneous according to the Brown-Forsythe test, the Mann-Whitney *U* test was used instead of the Student’s *t*-test. Among all obese groups of Zucker rats (OC, O + CO and O + CS group), significant differences were determined by one-factor analysis of variance (ANOVA) and Tukey’s *post hoc* test. If the data did not meet the assumptions mentioned previously, the Kruskal-Wallis ANOVA on ranks was used instead, followed by a Bonferroni-corrected Dunn’s *post hoc* test. The Pearson correlation coefficient was used to determine the relationship between selected variables. All findings were considered significant at *p* < 0.05, and all calculations were performed using Statistica version 13.1 (StatSoft Corp., Kraków, Poland).

**Fig. 2:**
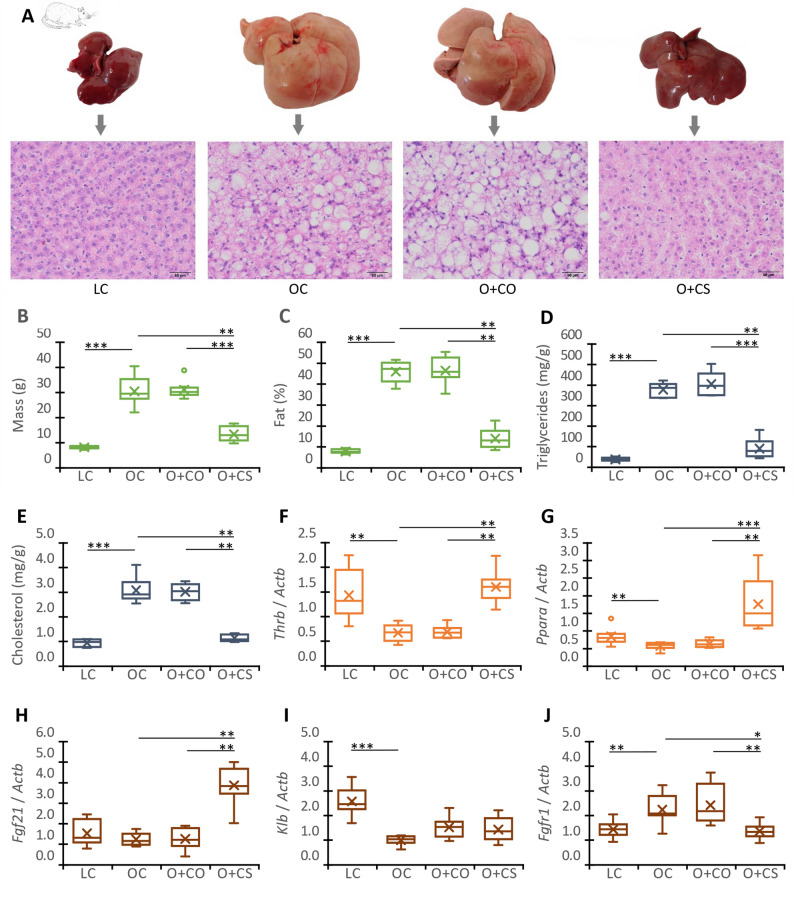
Dietary camelina seeds, but not their oil, counteract severe fatty liver and alter hepatic lipid metabolism in obese (*fa/fa*) Zucker rats. (**A**) Macroscopic and histological images of representative livers. (**B**,** C**) Liver mass and hepatic fat percentage. (**D**,** E**) Hepatic triglyceride and cholesterol contents. **F**,** G** Hepatic mRNA levels of thyroid hormone receptor *β* and peroxisome proliferator-activated receptor *α* genes (*Thrb* and *Ppara*), relative to the *β*-actin gene (*Actb*). (**H**,** J**) Hepatic mRNA levels of fibroblast growth factor 21, *β*-klotho and fibroblast growth factor receptor 1 genes (*Fgf21*, *Klb*, and *Fgfr1*). Data are presented as box-and-whisker plots for each group (*n* = 8): LC, lean control; OC, obese control; O + CO, obese fed a diet containing camelina seed oil; O + CS, obese fed a diet containing camelina seeds. An ‘×’ within each box indicates the mean value. Statistical significance: *p < 0.05, **p < 0.01, ***p < 0.001

**Fig. 3:**
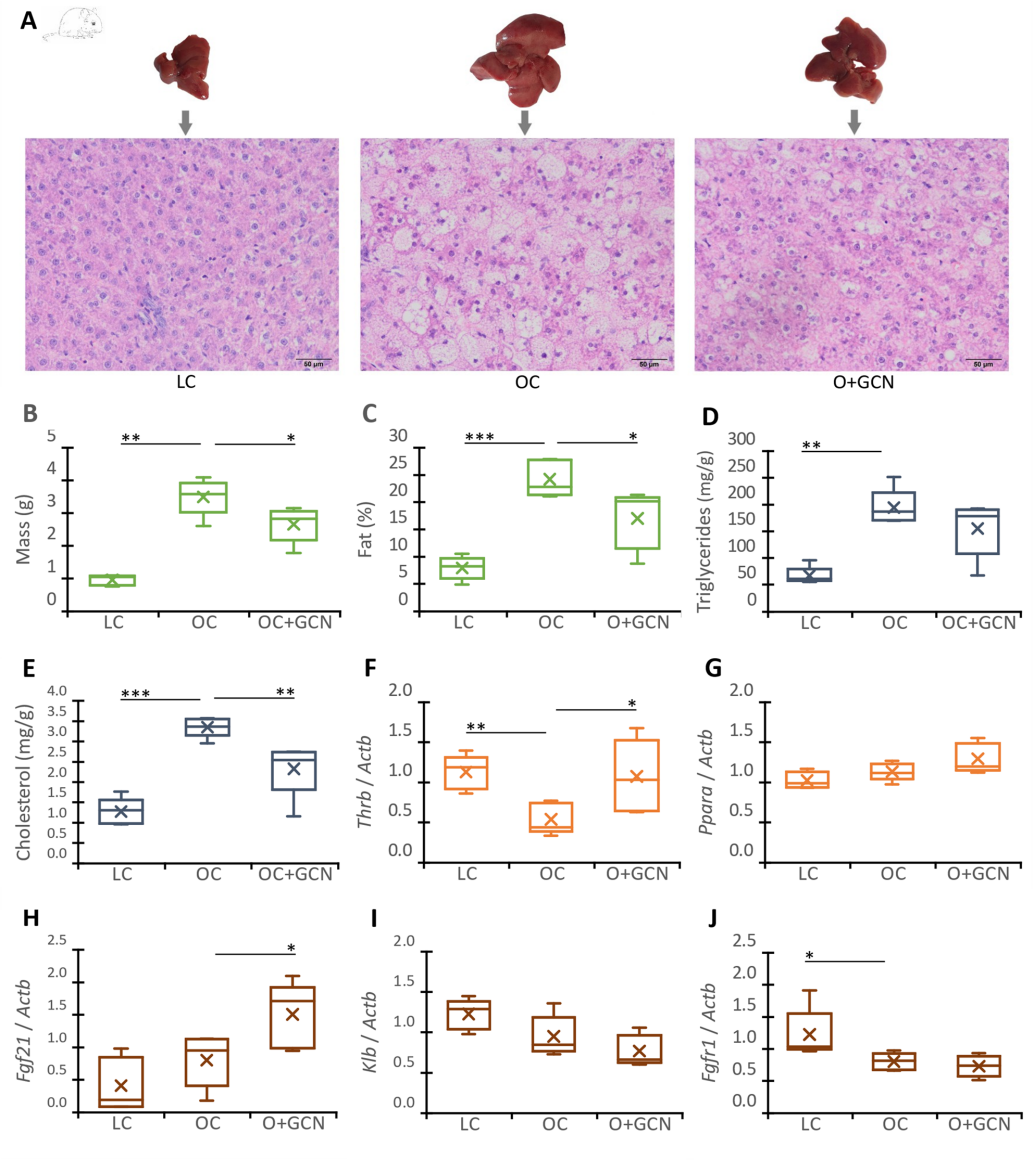
Oral glucocamelinin counteracts severe fatty liver and alters hepatic lipid metabolism in obese (*db/db*) mice. (**A**) Macroscopic and histological images of representative livers. (**B**,** C**) Liver mass and hepatic fat percentage. (**D**,** E**) Hepatic triglyceride and cholesterol contents. (**F**,** G**) Hepatic mRNA levels of thyroid hormone receptor *β* and peroxisome proliferator-activated receptor *α* genes (*Thrb* and *Ppara*), relative to the *β*-actin gene (*Actb*). (**H**,** J**) Hepatic mRNA levels of fibroblast growth factor 21, *β*-klotho and fibroblast growth factor receptor 1 genes (*Fgf21*, *Klb*, and *Fgfr1*). Data are presented as box-and-whisker plots for each group (*n* = 5): LC, lean control; OC, obese control; O + GCN, obese ingesting glucocamelinin. An ‘×’ within each box indicates the mean value. Statistical significance: *p < 0.05, **p < 0.01, ***p < 0.001

**Fig. 4:**
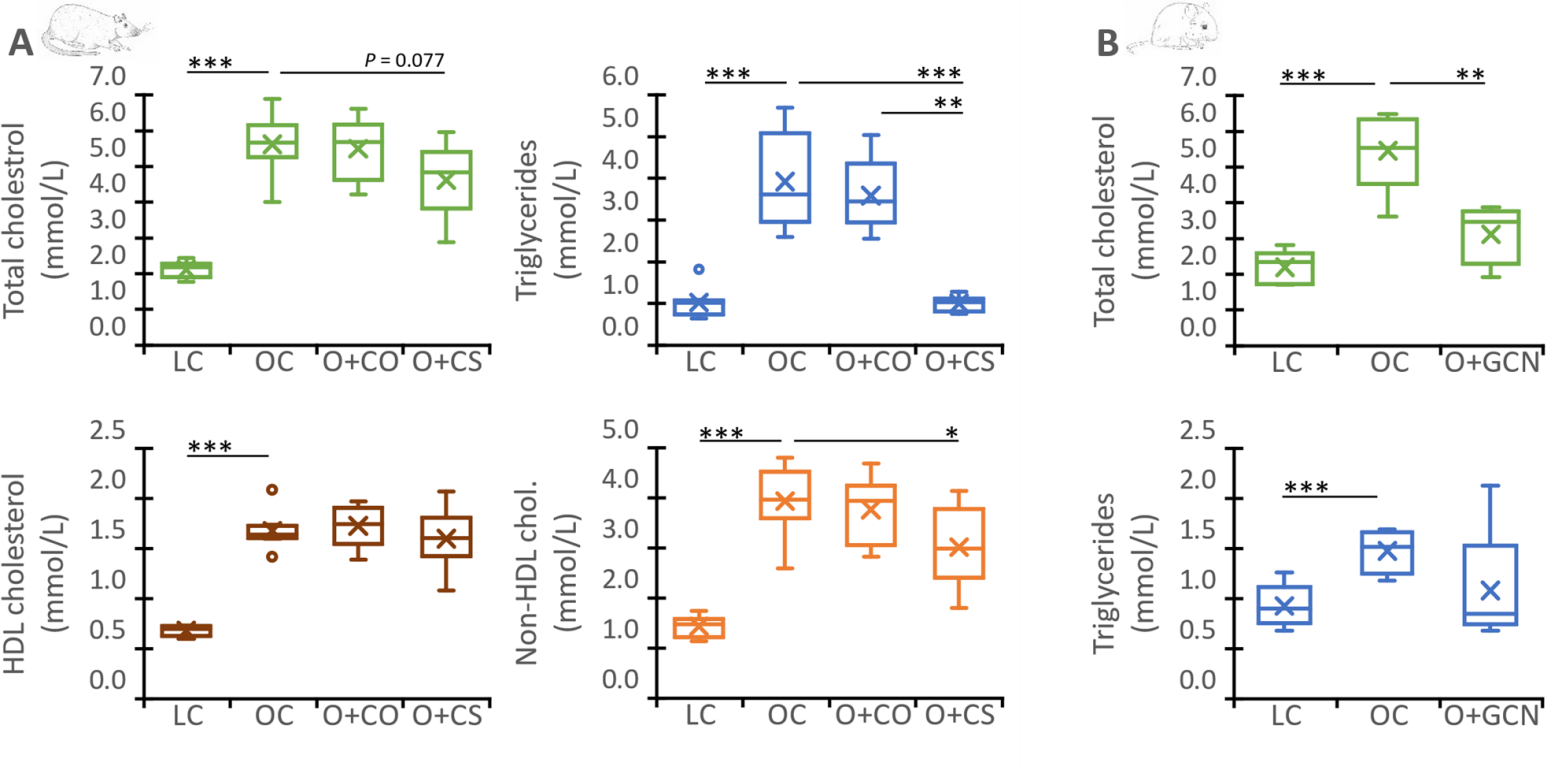
Dietary camelina seeds, but not their oil, and oral glucocamelinin affect the blood lipid profile in obese rodents. (**A**) Plasma concentrations of total cholesterol, triglyceride, HDL cholesterol and non-HDL cholesterol in obese (*fa/fa*) Zucker rats. Rat groups (*n* = 7–8): LC, lean control; OC, obese control; O + CO, obese fed a diet containing camelina seed oil; O + CS, obese fed a diet containing camelina seeds. (**B**) Plasma concentrations of total cholesterol and triglycerides in obese (*db/db*) mice. Mouse groups (*n* = 4–5): LC, lean control; OC, obese control; O + GCN, obese ingesting glucocamelinin. Statistical significance: *p < 0.05, **p < 0.01, ***p < 0.001. Data are presented as box-and-whisker plots. An ‘×’ within each box indicates the mean value

**Fig. 5:**
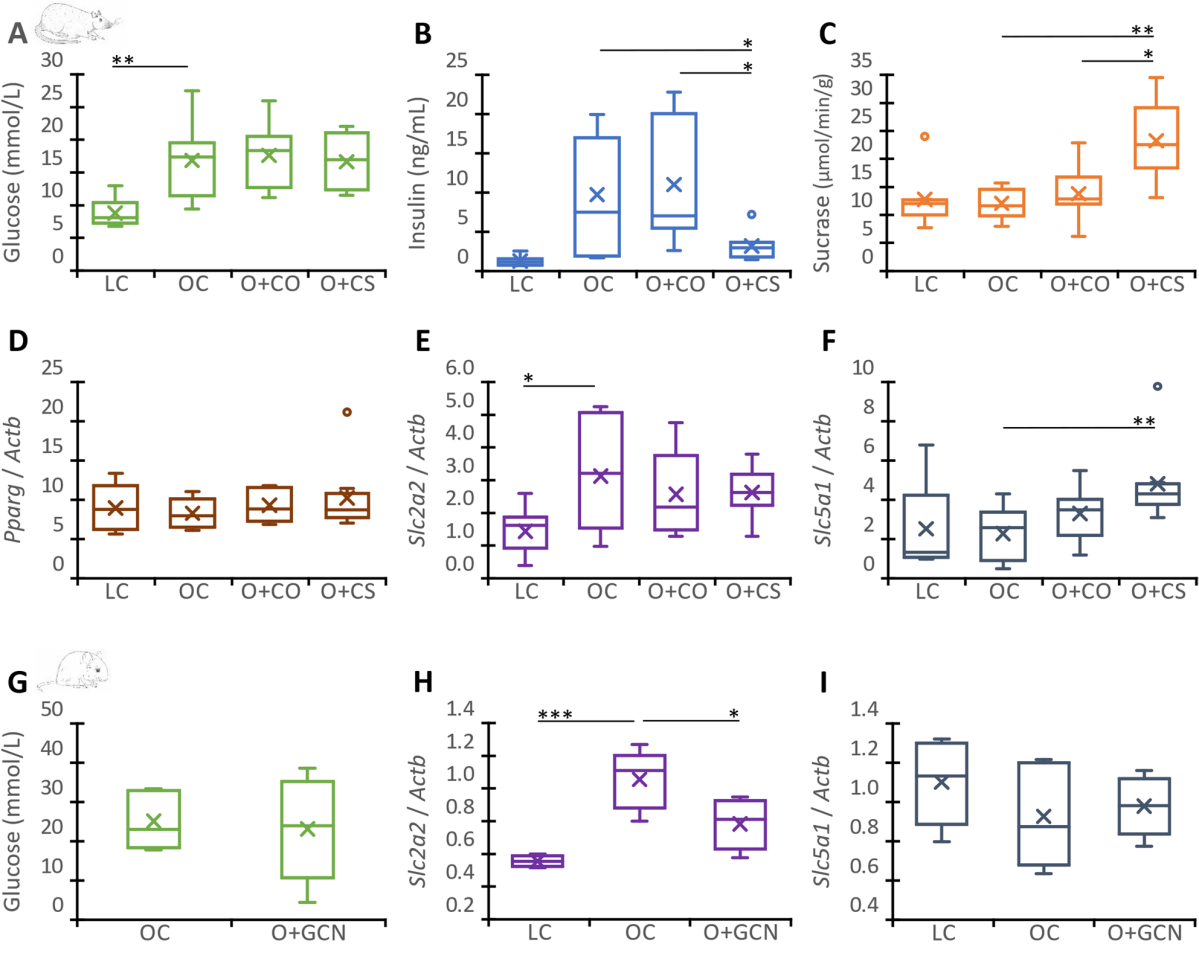
Effects of dietary camelina seeds and oral glucocamelinin on glucose metabolism in rodents: obese (*fa*/*fa*) Zucker rats (panels **A-F**) and obese (*db/db*) mice (panels **G-I**). (**A**,** B**) Glucose and insulin concentrations in blood plasma. **C** Sucrase activity in the small intestinal mucosa. (**D**) Hepatic mRNA level of the peroxisome proliferator-activated receptor gamma gene (*Pparg*), relative to the *β*-actin gene (*Actb*). (**E**,** F**) mRNA levels of the solute carrier family 2 member 2 gene (*Slc2a2*) and solute carrier family 5 member 1 gene (*Slc5a1*) in the small intestine. Rat groups (*n* = 7–8): LC, lean control; OC, obese control; O + CO, obese fed a diet containing camelina seed oil; O + CS, obese fed a diet containing camelina seeds. (**G**) Glucose concentration in blood plasma. (**H**,** I**) mRNA levels of *Slc2a2* and *Slc5a1* in the small intestine. Mouse groups (*n* = 5): LC, lean control; OC, obese control; O + GCN, obese ingesting glucocamelinin. Statistical significance: *p < 0.05, **p < 0.01, ***p < 0.001. Data are presented as box-and-whisker plots. An ‘×’ within each box indicates the mean value

In Figs. 2, 3 and 4, and 5, the results are presented as box-and-whisker plots. The lower and upper edges of the box represent the first and third quartiles, respectively, with the horizontal line inside the box indicating the median (second quartile). The whiskers extend to the minimum and maximum values, while individual points outside this range are shown as outliers. Additionally, an ‘×’ symbol inside the box marks the mean value.

## Results

### Effects of camelina seeds and their fractions on distal intestine and liver metabolism

In Experiment 1, we designed semipurified diets containing camelina seeds or camelina seed oil and fed them to obese Zucker rats to assess how certain seed fractions affect intestinal and liver functions and lipid metabolism in monogenic obesity. Recessive homozygotes of Zucker rats (*fa/fa*) have a mutation in the leptin receptor gene, so their food intake is not suppressed in the brain by leptin, leading to drastic weight gain associated with metabolic disorders, such as fatty liver, dyslipidemia, insulin resistance, and others [26]. In this experiment, camelina seeds contained high amounts of oil (36%), dietary fiber, and protein (26% both), and the proportions of fatty acids were very similar between their oil fraction and the corresponding cold-pressed oil (Table 1); in both cases, among others, the total PUFA contents were 55%-56% with *α*-linolenic acid (34%-35%) and linoleic acid (17% both) as the main essential *n-*3 and *n-*6 fatty acids, respectively. The diets were balanced in terms of macronutrients and fiber, and the percentage of oil from the tested seeds was the same in the diet with ground camelina seeds and in the diet with their cold-pressed oil (4% both; Fig. 1 and Supplementary Table 1). Thus, we were able to directly assess the effects of the oil fraction from the seeds, and indirectly, by comparing the obtained results, the effects of their non-oil fraction on the development of metabolic disorders.

After 5 weeks of experimental feeding, dietary camelina seeds and their oil (O + CS and O + CO groups, respectively) did not affect the diet intake or body weight of rats, which were comparable to those of obese control rats (group OC; Supplementary Table 2). Because camelina seeds are rich in fiber, we firstly examined their effects on short-chain fatty acid (SCFA) formation in the cecum, which, in rodents, is the main site for the breakdown of indigestible dietary components by bacteria. Some SCFAs, including acetate, propionate, and butyrate, are important mediators of distal intestine and liver functions [27], including lipid metabolism [28]; however, we did not find any significant effects of dietary camelina seeds on the formation of these fatty acids (Supplementary Table 3). What we did see was limited putrefaction due to dietary camelina seeds rather than their oil, which was evidenced by the lower cecal ammonia and branched SCFA concentrations (isobutyrate and isovalerate; O + CS group vs. the other obese groups; Supplementary Table 3), because these metabolites primarily originate from amino acid catabolism in the distal intestine [29, 30].

The most beneficial effects of dietary camelina seeds were seen in the liver, where they counteracted its significant hypertrophy and severe steatosis, which was confirmed by macroscopic and histological images and a several-fold lower content of total fat, triglycerides, and cholesterol in this organ (O + CS group vs. the other obese groups; Fig. 2A-E). Fatty liver disease is defined by the abundance of lipid droplets in hepatocytes [31], and in our study, the histological images clearly demonstrated numerous lipid droplets in the hepatocytes of obese rats (OC and O + CO groups), whereas dietary camelina seeds predominantly counteracted their occurrence (O + CS group; Fig. 2A). The liver function also improved, which was confirmed by the biochemical analysis of blood; the alanine transaminase and alkaline phosphatase activities and total bilirubin and bile acid concentrations were lower in rats fed a diet containing camelina seeds (O + CS vs. OC group; Supplementary Table 4). As importantly, dietary camelina oil did not significantly affect fatty liver itself or liver functions, indicating that the non-oil fraction of camelina seeds was responsible for all of the observed beneficial effects.

To identify potential seed components and mechanisms responsible for the observed counteraction of fatty liver, we further determined the hepatic mRNA expression of transcription factors involved in lipid metabolism [3]. Because camelina seed glucosinolates may affect the metabolism of thyroid hormones [14], we focused on thyroid hormone receptor *β* (THR-*β*), one of two isoforms predominating in the liver and encoded in rodents by the gene *Thrb* [32]. As a nuclear receptor, THR-*β* has been considered an important therapeutic target for lipid disorders and liver diseases [3, 32]. We found that *Thrb* was downregulated by the obesity itself in rats (OC vs. LC group), and dietary camelina seeds, but not their oil, upregulated it by more than twofold (O + CS group vs. the other obese groups, Fig. 2F). Additionally, the *Thrb* mRNA levels negatively correlated with the liver fat percentage in the O + CS group (*r* = − 0.782, *p* < 0.05; Supplementary Fig. 1A), suggesting that camelina glucosinolates counteract severe fatty liver through this receptor. Another nuclear receptor playing an important role in lipid metabolism is peroxisome proliferator-activated receptor *α* (PPAR-*α*), which, along with THR-*β*, belongs to the same family based on similarities in their ligand- and DNA-binding domains [33]. PPAR-*α*, encoded in rodents by the gene *Ppara*, regulates *β*-oxidation of fatty acids in the liver, among other functions [34]. *Ppara* was upregulated by more than twofold in the liver of obese rats fed camelina seeds compared to that in the livers of the other obese groups (Fig. 2G). However, in this case, *Ppara* mRNA levels did not correlate with the liver fat percentage (*r* = 0.010, *p* > 0.05; O + CS group; Supplementary Fig. 1A).

To further elucidate the mechanism underlying the counteraction of liver steatosis and other metabolic effects, we analyzed the hepatic mRNA expression of fibroblast growth factor 21 (FGF21) – a key metabolic regulator known to reduce hepatic lipid accumulation and enhance insulin sensitivity – along with its associated signaling components [3, 5]. Dietary camelina seeds upregulated the FGF21 gene (*Fgf21*) by more than twofold (O + CS group vs. all other groups, Fig. 2H), whereas the gene *Klb*, encoding *β*-klotho, was only downregulated by obesity itself (OC vs. LC group, Fig. 2I). *β*-klotho is an essential cofactor that enables FGF21 to bind to fibroblast growth factor receptor 1c (FGFR1c) and initiate downstream intracellular signaling pathways [5]. The gene *Fgfr1*, encoding FGFR1c, was upregulated in response to obesity and downregulated following dietary camelina seeds (Fig. 2J).

### Glucocamelinin in the counteraction of severe fatty liver

To confirm whether glucosinolates play a role in the beneficial effects of camelina seeds, we conducted Experiment 2 in obese (*db/db*) mice (Fig. 1), whose genetic background of obesity is analogous to that of obese (*fa/fa*) Zucker rats [26]. We chose glucocamelinin, the main glucosinolate of camelina seeds [16, 20], and administered it orally to obese mice once a day. The daily dose of glucocamelinin was similar to the daily dose of glucosinolates ingested by the rats together with camelina seeds in Experiment 1.

After 5 weeks of oral administration, glucocamelinin (O + GCN group) did not affect dietary intake, body weight, or body composition (fat and lean percentages), which were comparable to those of obese control mice (OC group; Supplementary Table 5). These results, together with findings from Experiment 1, allow us to conclude that feeding obese rodents with camelina seeds and camelina-specific glucosinolates does not affect the development of monogenic obesity itself (Supplementary Tables 2 and 5). As in Experiment 1, oral glucocamelinin significantly counteracted liver hypertrophy and lipid accumulation, which was confirmed by macroscopic and histological images and by lower total fat and cholesterol contents in this organ (OC vs. O + GCN group; Fig. 3A-E). However, these beneficial differences were slightly less significant than those seen following dietary camelina seeds.

Oral glucocamelinin also upregulated *Thrb* in the liver but did not affect the expression of *Ppara* (O + GCN vs. OC group; Fig. 3F-G). As in Experiment 1, only *Thrb* showed a negative correlation with liver fat percentage in the O + GCN group (*r* = − 0.900, *p* < 0.05; Supplementary Fig. 1B), suggesting that camelina seed glucosinolates counteract severe fatty liver through THR-*β*. Regarding the FGF21 signaling pathway, oral glucocamelinin also upregulated hepatic expression of *Fgf21* (O + GCN vs. OC group; Fig. 3H) and did not affect hepatic expression of *Klb* (Fig. 3I). The hepatic expression of *Fgfr1* was downregulated in response to obesity (Fig. 3J).

Furthermore, we excluded oral glucocamelinin as a factor affecting SCFA formation in the cecum (Experiment 2; Supplementary Table 6), including isobutyrate and isovalerate, which are of putrefactive origin [30]. This finding apparently indicates that the reduced putrefaction seen in the cecum of obese rats (Experiment 1; Supplementary Table 3) was due to other components of the non-oil fraction of camelina seeds, such as fiber or protein.

### Effects of camelina seeds and glucocamelinin on blood lipid profile

In addition to counteracting fatty liver, dietary camelina seeds, but not their oil fraction, and oral glucocamelinin also affected the blood lipid profile in genetically obese rodents. Obese control rats and mice had close to or more than twice the blood cholesterol concentration of their lean counterparts (Experiments 1 and 2; respective OC vs. respective LC groups; Fig. 4), which was attenuated by both experimental factors. In obese rats fed with camelina seeds, the total cholesterol and non-HDL cholesterol concentration tended to be lower and were significantly lower, respectively, compared to the obese control group (Fig. 4A). In obese mice, in turn, the total cholesterol-lowering effect of glucocamelinin was even more significant (O + GCN vs. OC group; Fig. 4B). The available literature indicates that certain selective THR-*β* agonists can lower blood cholesterol [35, 36], which is also the case for well-known cholesterol-lowering drugs, such as statins, that can indirectly activate PPAR-*α* and the expression of its gene [37]. In both obese rodent species, we additionally found higher blood triglyceride concentrations than in their lean counterparts (respective LC vs. OC groups; Fig. 4). Dietary camelina seeds (OC vs. O + CS group; Fig. 4A), but not their oil, and, to a lesser extent, oral glucocamelinin, counteracted this effect.

### Effects of camelina seeds and glucocamelinin on glucose metabolism

A very important problem with THR-*β* agonists tested so far is that they disturb glucose metabolism while counteracting hepatic steatosis [38]. We did not find any effect of dietary camelina seeds on blood glucose concentration, which was only higher due to the obesogenic state of rats itself (Experiment 1; OC vs. LC group; Fig. 5A), and the blood glucose concentration was also comparable between both groups of obese mice (Experiment 2; OC vs. O + GCN; Fig. 5G). Dietary camelina seeds also did not affect hepatic mRNA levels of the gene *Pparg* (Experiment 1; Fig. 5D) encoding peroxisome proliferator-activated receptor gamma (PPAR-*γ*), one of the transcription factors involved in the regulation of glucose metabolism in the body [34]. Instead, a lower blood insulin concentration was observed in obese rats receiving camelina seeds (O + CS vs. OC and O + CO groups; Fig. 5B).

The mucosal sucrase activity in the small intestine of obese rats fed dietary camelina seeds was nearly twice as high as that in the other groups (Experiment 1; Fig. 5C), which was not the case for maltase and lactase activities (Supplementary Table 7). Additionally, the small intestinal mass relative to body weight was also higher in obese rats fed with camelina seeds (Supplementary Table 7), which was mainly due to excess digesta remaining in the ileal lumen. According to Zubr et al. [39], camelina seeds contain a relatively high amount of sucrose (5.5%), the presence of which was not strictly controlled in the rats’ diet. This may explain the increased sucrase activity in response to the substrate.

In the small intestine of obese rats fed dietary camelina seeds, we also noticed a higher mRNA level of the solute carrier family 5 member 1 gene (*Slc5a1*) encoding sodium-dependent glucose cotransporter 1 (SGLT1, O + CS vs. OC group; Fig. 5F), which mediates active glucose absorption in enterocytes [40]. The main transporter responsible, in turn, for facilitative glucose absorption in enterocytes is glucose transporter 2 (GLUT2) [40], but the mRNA level of the solute carrier family 2 member 2 gene (*Slc2a2*) encoding this transporter was only affected by the obesogenic state itself in rats (LC vs. OC group; Fig. 5E). Since SGLT1 had been identified as a carrier for some glucose-containing phytochemicals [41, 42], we speculated about the involvement of this cotransporter in the absorption of glucosinolates, which also contain glucose in their structure. However, oral glucocamelinin did not affect the *Slc5a1* mRNA level in the small intestine of obese mice (O + GCN vs. OC group, Fig. 5I), indicating that other components of camelina seeds were responsible for this upregulation. What we did see in Experiment 2, conversely, was that oral glucocamelinin downregulated *Slc2a2* (O + GCN vs. OC group), which, in turn, was upregulated by the obesogenic state itself in mice (OC vs. LC group, Fig. 5H).

## Discussion

The aim of this research was to verify whether camelina seeds can attenuate disorders specific to monogenic obesity, including a severe form of fatty liver, and which seed components are responsible for these benefits. The camelina seeds and oil used were very rich in PUFAs (55–56% of total fat), especially in *α*-linolenic acid (34%-35% of total fat) representing *n*-3 fatty acids, which are nutrients that attenuate inflammation and improve lipid metabolism by stimulating *β*-oxidation of fatty acids [43]. However, they also contained some amounts of erucic acid (approx. 3% of total fat), an *n-*9 monounsaturated fatty acid (MUFA), which causes lipid disorders at high and chronic exposure, including myocardial lipidosis, due to its poor *β*-oxidation in mitochondria [44]. Although we did not observe any alterations in heart mass in response to the dietary interventions (data not shown), this might be a partial explanation for why camelina seed oil did not significantly affect any of the measured indices of lipid metabolism in obese rats. In addition to the opposing effects of camelina seed fatty acids, the metabolic disorders observed in monogenic obesity may have been too severe to be attenuated by dietary PUFAs alone. In an experiment on rats with diabetes induced by a high-fat diet and streptozotocin injection, camelina oil improved liver function only when combined with high-intensity interval training [45]. A meta-analysis of clinical trials confirms a cholesterol-lowering effect of camelina oil only at doses lower than 30 mg/day and in treatments lasting more than 8 weeks [46].

The design of Experiment 1 (see Materials and methods and Fig. 1 for details) allowed direct assessment of the oil fraction and, by comparing the O + CO and O + CS groups, indirect assessment of the non-oil fraction of camelina seeds in relation to the development of metabolic disorders. We thus concluded that most of the beneficial effects of camelina seeds, including the counteraction of hepatic steatosis, were due to their non-oil fraction, consisting of dietary fiber, among other components (Table 1). However, the non-oil fraction did not influence major bacterial metabolites of dietary fiber in the distal intestine (acetate, propionate, butyrate), which are mediators of intestinal and hepatic functions [27]. Given that the beneficial effects could not be explained by fiber-derived metabolites, we focused on glucosinolates, which are present in relatively high amounts in dry camelina seeds, approximately 65% of which is glucocamelinin [20], and whose content in the press cake may be more than twice as high [16]. This is consistent with the fact that glucosinolates are highly hydrophilic and have a negative octanol–water partition coefficient [47]. We therefore selected glucocamelinin for further investigation and found that its oral administration also attenuated severe fatty liver. However, this effect was slightly less pronounced than that of dietary camelina seeds, probably due to a different methodological approach in the experiments on rats and mice (continuous dietary supplementation vs. once-daily oral administration constrained by limited glucocamelinin availability) and/or the absence of other bioactive compounds present in the non-oil fraction of camelina seeds. Specifically, in obese rats, both total fat percentage and triglyceride contents in the liver were lower following dietary camelina seeds, whereas in obese mice, the total fat percentage was also lower and the triglyceride contents tended to be lower following oral glucocamelinin (Fig. 2C and D vs. Figure 3C and D). These results suggest that other important liver lipids, such as free fatty acids, diacylglycerols, or phospholipids [48], may have been decreased in obese mice. Further animal studies on diet-induced obesity, as well as clinical trials, are needed to verify the beneficial effects of camelina seeds and glucocamelinin, and to exclude any potential side effects associated with the ingestion of camelina seed components.

The rodent model of fatty liver used in this study adequately mimics MASLD, the current term for the condition previously known as non-alcoholic fatty liver disease (NAFLD) [26, 49]. However, we refrain from using this term when describing our findings in order to clearly distinguish experimental models from clinical cases. Nevertheless, there are several classes of drugs under development that target MASLD and its more advanced stages, especially ligands (agonists or antagonists) of various nuclear receptors, such as PPARs, farnesoid X receptor, and THR-*β* [50]. These nuclear receptors are transcriptional regulators of key metabolic processes in the liver, such as lipid and glucose metabolism, energy expenditure, and bile acid homeostasis, as well as processes associated with MASLD progression, such as inflammation and fibrosis [51]. THR-*β* has two isoforms, *β*1 and *β*2, which are splice variants of the same gene [38]. THR-*β*1 is the predominant isoform in the liver (80% of all isoforms), and its activation stimulates *β*-oxidation of fatty acids and the excretion of cholesterol and phospholipids into the bile [32, 51]. Here, we found that the mechanism behind the action of both camelina seeds and glucocamelinin may involve activation of the gene encoding THR-*β* in the liver. However, THR-*β* activation can also be associated with side effects, especially disorders in glucose metabolism, as demonstrated in experiments on rats for two THR-*β* agonists [38]. Our study indicates that glucocamelinin does not disrupt glucose metabolism, and that camelina seeds may even reduce hyperinsulinemia. A further mechanistic explanation for the reduced hepatic lipid accumulation and reduced hyperinsulinemia observed in this study seems to be the THR-*β*–mediated stimulation of FGF21 signaling. FGF21 analogues are promising therapeutic candidates for the treatment of MASLD, type 2 diabetes and obesity [50]. In addition to PPAR-*α*, THR-*β* is recognized as a key transcription factor that directly stimulates hepatic FGF21 expression [3, 52].

The conducted experiments have certain limitations that should be taken into account in future studies, including those focusing on dose–response relationships. Thyroid function was not assessed, which is of significant importance in the context of the potential risk that glucosinolates may pose to this organ and may represent a possible side effect of the proposed intervention. Due to the limited availability of glucocamelinin in the form of chromatographic standard, the experiments in mice were conducted on a relatively small number of animals. Furthermore, due to the lack of drugs specifically dedicated to the treatment of MASLD, a positive control was not included, which could have served as a valuable reference point. Future studies should also include a detailed histopathological analysis of the liver. Such an analysis, in combination with hepatic lipid content and plasma markers of liver function determined in this study, would enable the evaluation of additional structural and inflammatory changes, thereby providing a more complete picture of the intervention’s effects.

## Conclusions

This study demonstrated that dietary inclusion of camelina seeds counteracts metabolic disorders in rats with monogenic obesity, particularly the development of severe fatty liver. Dietary camelina seeds are also able to reduce putrefactive processes in the distal intestine and attenuate hyperinsulinemia in these animals. The beneficial effects on the liver are attributed to the non-oil fraction of the seeds, as shown in the rat model, while glucocamelinin is an important bioactive compound contributing to these effects, as shown in mice with the same obesity model. The observed effects may involve hepatic activation of FGF21 signaling through THR-β, which requires further investigation. Overall, these findings suggest that camelina seeds may be considered both a dietary component and a source of bioactive compounds relevant to MASLD, supporting the rationale for further research.

## Supplementary Information

Below is the link to the electronic supplementary material.

Supplementary Material 1

## Data Availability

No datasets were generated or analysed during the current study.
