# Supplementary material for: Camelina seeds and their glucosinolate, glucocamelinin, counteract severe fatty liver in rodents with monogenic obesity
Source: Genes Nutr. 2026 Jun 6;21:19. doi: 10.1186/s12263-026-00807-x (PMC13459901; doi:10.1186/s12263-026-00807-x)
Supplement: Supplementary file 1 [file 12263_2026_807_MOESM1_ESM.docx]

Supporting information

**Camelina seeds and their glucosinolate, glucocamelinin, counteract severe fatty liver in rodents with monogenic obesity**

Adam Jurgoński et al.

Correspondence: a.jurgonski@pan.olsztyn.pl

TABLE OF CONTENTS

Supplementary Figure 1	p. 2

Supplementary Table 1	p. 3

Supplementary Table 2	p. 4

Supplementary Table 3	p. 5

Supplementary Table 4	p. 6

Supplementary Table 5	p. 7

Supplementary Table 6	p. 8

Supplementary Table 7	p. 9

Supplementary Figure 1. Correlation between hepatic fat content and mRNA levels of selected transcription factors in rodents: obese (*fa*/*fa*) Zucker rats (panel **A**) and obese (*db/db*) mice (panel **B**). *Thrb*, thyroid hormone receptor *β* gene; *Ppara*, peroxisome proliferator-activated receptor *α* gene; *Actb*, *β*-actin gene. Statistical significance: **p* < 0.05.

Supplementary Table 1. Composition of the diets for Zucker rats.

|  | | | | **Group^1^** | | | |
| --- | --- | --- | --- | --- | --- | --- | --- |
|  |  |  |  | **LC** | **OC** | **O+CO** | **O+CS** |
| Ingredients (%) | | | |  |  |  |  |
|  | Casein^2^ | | | 20.00 | 20.00 | 20.00 | 16.76 |
|  | DL-methionine | | | 0.3 | 0.3 | 0.3 | 0.3 |
|  | Rapeseed oil^3^ | | | 4 | 4 | 4 | 4 |
|  | Palm oil^4^ | | | 4 | 4 | – | – |
|  | Camelina seed oil^5^ | | | – | – | 4 | – |
|  | Camelina seeds^5^ | | | – | – | – | 11.23 |
|  | Corn starch | | | 52.00 | 52.00 | 52.00 | 50.91 |
|  | Sucrose | | | 10 | 10 | 10 | 10 |
|  | Cellulose | | | 5.00 | 5.00 | 5.00 | 2.10 |
|  | Mineral mix^6^ | | | 3.5 | 3.5 | 3.5 | 3.5 |
|  | Vitamin mix^6^ | | | 1 | 1 | 1 | 1 |
|  | Choline chloride | | | 0.2 | 0.2 | 0.2 | 0.2 |
| Nutrients (%) | | | |  |  |  |  |
|  | Protein | | | 18.0 | 18.0 | 18.0 | 18.0 |
|  | Fat | | | 8.3 | 8.3 | 8.3 | 8.3 |
|  |  | SFAs^7^ | | 2.11 | 2.11 | 1.42 | 1.44 |
|  |  | MUFAs^7^ | | 4.09 | 4.09 | 3.23 | 3.25 |
|  |  | PUFAs^7^ | | 1.40 | 1.40 | 2.94 | 2.89 |
|  |  |  | *n*-3 | 0.33 | 0.33 | 1.63 | 1.58 |
|  |  |  | *n*-6 | 1.08 | 1.08 | 1.32 | 1.31 |
|  | Fiber | | | 5.0 | 5.0 | 5.0 | 5.0 |

^1^ LC, lean control; OC, obese control; O+CO, obese fed a diet containing camelina seed oil; O+CS, obese fed a diet containing camelina seeds.

^2^ Casein preparation (g/100 g): crude protein, 88.70; crude fat, 0.3; ash, 2.0; water, 8.0.

^3^ Canola type oil with the following fatty acid profile (%): SFAs, 6.49; MUFAs, 62.9 including oleic acid, 56.9; PUFAs, 25.9 including linoleic acid and α-linolenic acid, 17.5 and 8.06, respectively.

^4^ Fatty acid profile (%): SFAs, 46.3 including palmitic acid, 40.8; MUFAs, 39.4 including oleic acid, 39.1; PUFAs, 9.21 including linoleic acid, 9.1.

^5^ Chemical composition in Table 1 (main article).

^6^ Recommended for the AIN-93G diet.

^7^ MUFAs, monounsaturated fatty acids; PUFAs, polyunsaturated fatty acids; SFAs, saturated fatty acid.

Supplementary Table 2. Dietary camelina seeds and their oil do not affect the body weight and dietary intake of obese (*fa/fa*) Zucker rats^1^.

|  | **Group^2^** | | | |
| --- | --- | --- | --- | --- |
|  | **LC** | **OC** | **O+CO** | **O+CS** |
| Initial body weight (g) | 134±0.931^*^ | 191±5.62 | 194±5.56 | 192±6.85 |
| Dietary intake (g/day) | 12.3±0.244^*^ | 18.9±0.771 | 19.3±0.614 | 17.3±1.14 |
| Final body weight (g) | 203±3.34^*^ | 322±10.82 | 338±11.6 | 314±15.7 |
| Body weight gain (g) | 69.1±3.73^*^ | 131±6.48 | 145±7.01 | 122±10.6 |

^1^ Values are means ± SEMs (*n* = 8 / group).

^2^ LC, lean control; OC, obese control; O+CO, obese fed a diet containing camelina seed oil; O+CS, obese fed a diet containing camelina seeds.

^*^ *p* < 0.05 vs. OC group (Student’s *t*-test or Mann-Whitney *U* test).
Supplementary Table 3. Dietary camelina seeds affect metabolites of putrefactive origin in the distal intestine of obese (*fa/fa*) Zucker rats^1^.

|  | | **Group^2^** | | | |
| --- | --- | --- | --- | --- | --- |
|  |  | **LC** | **OC** | **O+CO** | **O+CS** |
| Cecum wall (g/100 g) | | 0.185±0.010^*^ | 0.101±0.007^b^ | 0.099±0.004^b^ | 0.155±0.007^a^ |
| Digesta (g/g cecum) | | 3.79±0.155^*^ | 2.74±0.217 | 2.56±0.182 | 2.68±0.137 |
| pH of cecal digesta | | 7.37±0.047^*^ | 7.82±0.085 | 7.79±0.106 | 7.59±0.061 |
| Ammonia (mg/g cecal digesta) | | 0.199±0.011^*^ | 0.256±0.005^a^ | 0.213±0.016^ab^ | 0.203±0.005^b^ |
| SCFA (µmol/g cecal digesta)^5^ | |  |  |  |  |
|  | Acetate | 38.2±0.451 | 36.4±2.64 | 36.8±3.52 | 30.9±1.17 |
|  | Propionate | 11.1±0.720 | 9.91±0.323 | 11.5±1.33 | 11.6±0.562 |
|  | Isobutyrate | 1.14±0.065 | 1.07±0.090^a^ | 1.08±0.115^a^ | 0.708±0.045^b^ |
|  | Butyrate | 7.16±0.420^*^ | 4.64±0.354 | 4.34±0.489 | 3.47±0.399 |
|  | Isovalerate | 1.14±0.071 | 1.10±0.113^a^ | 0.967±0.097^ab^ | 0.671±0.056^b^ |
|  | Valerate | 1.28±0.077 | 1.10±0.054^a^ | 1.06±0.122^ab^ | 0.783±0.053^b^ |
|  | Total | 60.1±1.28 | 54.2±3.029 | 55.7±5.18 | 48.2±2.10 |
| SCFAs (µmol/cecal digesta mass)^5^ | | 85.4±5.51^*^ | 47.1±5.16 | 48.4±6.45 | 61.2±2.47 |

^1^ Values are means ± SEMs (*n* = 8 / group).

^2^ LC, lean control; OC, obese control; O+CO, obese fed a diet containing camelina seed oil; O+CS, obese fed a diet containing camelina seeds.

^*^ *p* < 0.05 vs. OC group (Student’s *t*-test or Mann-Whitney *U* test).

^a,b^ *p* < 0.05 if labelled means in a row do not share the same letter (Tukey’s or Dunn’s *post hoc* test preceded by one-factor ANOVA or one-factor ANOVA on ranks).

^5^ SCFAs, short-chain fatty acids.
Supplementary Table 4. Dietary camelina seeds, but not their oil, improve liver functions in obese (*fa/fa*) Zucker rats^1^.

|  | **Group^2^** | | | |
| --- | --- | --- | --- | --- |
|  | **LC** | **OC** | **O+CO** | **O+CS** |
| AST (U/L)^3^ | 59.7±2.07^*^ | 390±80.0^a^ | 348±17.9^a^ | 105±18.2^b^ |
| ALT (U/L)^3^ | 24.9±1.94^*^ | 101±9.31^a^ | 82.2±3.57^ab^ | 64.5±4.80^b^ |
| ALP (U/L)^3^ | 239±17.1^*^ | 703±62.3^a^ | 817±71.1^a^ | 216±16.4^b^ |
| Total bilirubin (µmol/L) | <DL^4^ | 11.2±2.23 | 8.03±1.58 | <DL^4^ |
| Albumin (µmol/L) | 317±7.25^*^ | 137±3.83^b^ | 144±4.27^b^ | 235±20.1^a^ |
| Total bile acids (µmol/L) | 24.0±3.47^*^ | 51.3±8.35^a^ | 46.8±5.30^a^ | 24.0±0.651^b^ |

^1^ Values are means ± SEMs (*n* = 7–8 / group).

^2^ LC, lean control; OC, obese control; O+CO, obese fed a diet containing camelina seed oil; O+CS, obese fed a diet containing camelina seeds.

^*^ *p* < 0.05 vs. OC group (Student’s *t*-test or Mann-Whitney *U* test).

^a,b^ *p* < 0.05 if labelled means in a row do not share the same letter (Tukey’s or Dunn’s *post hoc* test preceded by one-factor ANOVA or one-factor ANOVA on ranks).

^3^ AST, aspartate transaminase; ALT, alanine transaminase; ALP, alkaline phosphatase.

^4^ DL, detection limit = 2.90 µmol/L.
Supplementary Table 5. Oral glucocamelinin does not affect the dietary intake and body composition of obese (*db/db*) mice^1^.

|  | | | **Group^2^** | | |
| --- | --- | --- | --- | --- | --- |
|  |  |  | **LC** | **OC** | **O+GCN** |
| Initial body weight (g) | | | 20.9±0.344^*^ | 35.4±2.84 | 35.1±2.23 |
|  | Initial fat (%) | | 17.7±0.683^*^ | 55.2±4.29 | 53.6±2.61 |
|  | Initial lean (%) | | 68.6±0.682^*^ | 35.7±3.08 | 37.0±2.14 |
| Dietary intake (g/day) | | | 3.38±0.092^*^ | 5.25±0.122 | 6.32±0.816 |
| Final body weight (g) | | | 23.4±0.427^*^ | 44.0±2.20 | 43.1±1.98 |
|  | | Final fat (%) | 21.1±3.30^*^ | 60.4±2.52 | 61.9±1.60 |
|  | | Final lean (%) | 60.8±2.94^*^ | 28.2±2.06 | 26.8±1.33 |
| Body weight gain (g) | | | 2.45±0.315^*^ | 8.56±0.906 | 8.07±1.58 |

^1^ Values are means ± SEMs (*n* = 5 / group).

^2^ LC, lean control; OC, obese control; O+GCN, obese ingesting glucocamelinin.

^*^ *p* < 0.05 vs. OC group (Student’s *t*-test or Mann-Whitney *U* test).
Supplementary Table 6. Oral glucocamelinin does not affect metabolite formations in the distal intestine of obese (*db/db*) mice^1^.

|  | | **Group^2^** | | |
| --- | --- | --- | --- | --- |
|  |  | **LC** | **OC** | **O+GCN** |
| Cecum wall (g/100 g) | | 0.573±0.056 | 0.403±0.035 | 0.514±0.059 |
| Digesta (g/g cecum) | | 2.68±0.193 | 2.71±0.170 | 2.78±0.356 |
| pH of cecal digesta | | 7.08±0.138 | 7.08±0.155 | 6.82±0.093 |
| SCFA (µmol/g cecal digesta) | |  |  |  |
|  | Acetate | 70.2±4.19 | 59.3±5.51 | 51.9±5.59 |
|  | Propionate | 8.09±0.632 | 8.32±1.141 | 6.73±0.525 |
|  | Isobutyrate | 0.438±0.078 | 0.509±0.073 | 0.549±0.081 |
|  | Butyrate | 22.8±6.38 | 23.9±2.01 | 25.1±4.36 |
|  | Isovalerate | 0.403±0.070 | 0.475±0.074 | 0.529±0.092 |
|  | Valerate | 0.731±0.108 | 0.827±0.092 | 0.697±0.059 |
|  | Total | 103±8.42 | 93.3±7.74 | 85.5±9.57 |
| SCFAs (µmol/cecal digesta mass) | | 35.5±2.09 | 45.0±6.62 | 49.7±5.90 |

^1^ Values are means ± SEMs (*n* = 5 / group).

^2^ LC, lean control; OC, obese control; O+GCN, obese ingesting glucocamelinin.

^3^ SCFAs, short-chain fatty acids.
Supplementary Table 7. Effects of dietary camelina seeds on maltase and lactase activity in the small intestine of obese (*fa/fa*) Zucker rats^1^.

|  | **Group^2^** | | | |
| --- | --- | --- | --- | --- |
|  | **LC** | **OC** | **O+CO** | **O+CS** |
| Small intestine (g/100 g b.wt) | 2.38±0.055^*^ | 1.79±0.054^b^ | 1.73±0.038^b^ | 2.34±0.054^a^ |
| Maltase (µmol/min/g) | 104±15.6^*^ | 78.2±7.47 | 88.1±9.53 | 108±38.2 |
| Lactase (µmol/min/g) | 5.36±0.649 | 4.06±0.309 | 4.22±0.413 | 4.63±0.470 |

^1^ Values are means ± SEMs (*n* = 8 / group).

^2^ LC, lean control; OC, obese control; O+CO, obese fed a diet containing camelina seed oil; O+CS, obese fed a diet containing camelina seeds.

^*^ *p* < 0.05 vs. OC group (Student’s *t*-test or Mann-Whitney *U* test).

^a,b^ *p* < 0.05 if labelled means in a row do not share the same letter (Tukey’s or Dunn’s *post hoc* test preceded by one-factor ANOVA or one-factor ANOVA on ranks).
